# Identifying SARS-CoV Membrane Protein Amino Acid Residues Linked to Virus-Like Particle Assembly

**DOI:** 10.1371/journal.pone.0064013

**Published:** 2013-05-20

**Authors:** Ying-Tzu Tseng, Chia-Hui Chang, Shiu-Mei Wang, Kuo-Jung Huang, Chin-Tien Wang

**Affiliations:** 1 Department of Medical Research and Education, Taipei Veterans General Hospital, Taipei, Taiwan; 2 Institute of Public Health, National Yang-Ming University School of Medicine, Taipei, Taiwan; 3 Institute of Clinical Medicine, National Yang-Ming University School of Medicine, Taipei, Taiwan; German Primate Center, Germany

## Abstract

Severe acute respiratory syndrome coronavirus (SARS-CoV) membrane (M) proteins are capable of self-assembly and release in the form of membrane-enveloped vesicles, and of forming virus-like particles (VLPs) when coexpressed with SARS-CoV nucleocapsid (N) protein. According to previous deletion analyses, M self-assembly involves multiple M sequence regions. To identify important M amino acid residues for VLP assembly, we coexpressed N with multiple M mutants containing substitution mutations at the amino-terminal ectodomain, carboxyl-terminal endodomain, or transmembrane segments. Our results indicate that a dileucine motif in the endodomain tail (218LL219) is required for efficient N packaging into VLPs. Results from cross-linking VLP analyses suggest that the cysteine residues 63, 85 and 158 are not in close proximity to the M dimer interface. We noted a significant reduction in M secretion due to serine replacement for C158, but not for C63 or C85. Further analysis suggests that C158 is involved in M-N interaction. In addition to mutations of the highly conserved 107-SWWSFNPE-114 motif, substitutions at codons W19, W57, P58, W91, Y94 or F95 all resulted in significantly reduced VLP yields, largely due to defective M secretion. VLP production was not significantly affected by a tryptophan replacement of Y94 or F95 or a phenylalanine replacement of W19, W57 or W91. Combined, these results indicate the involvement of specific M amino acids during SARS-CoV virus assembly, and suggest that aromatic residue retention at specific positions is critical for M function in terms of directing virus assembly.

## Introduction

The highly contagious severe acute respiratory syndrome (SARS) affected individuals in 30 countries in 2002 and 2003 [1]. Its causative agent was identified as a novel SARS-associated coronavirus (SARS-CoV) [1], [2], [3] that was initially classified as part of a separate coronavirus group [4], [5], [6], [7], but is now described as a betacoronavirus [8]. As with most coronaviruses, SARS-CoV encodes four structural proteins: spike (S), membrane (M), envelope (E) and nucleocapsid (N) [4], [9]. Mature coronavirus particle assembly involves protein-protein and protein-RNA interactions. M, the most abundant structural protein [10], is thought to play a central role in directing virus assembly and budding via interaction with E, S and N [10],[11],[12],[13],[14],[15],[16],[17],[18]. Translated on free polysomes, N is associated with newly synthesized viral genomic RNA to form helical nucleocapsids [19]. The M membrane glycoprotein is co-translationally inserted into the endoplasmic reticulum (ER) and transported to Golgi complexes [20], [21]. M interacts with nucleocapsids on the cell membranes of ER or Golgi complexes [22], [23], [24], [25], [26]. In a similar manner, S and E proteins are translated on membrane-bound polysomes, inserted into the ER, and transported to Golgi complexes, where E and M interact and trigger virion budding with enclosed nucleocapsids [14], [19]. S is incorporated into virions via interactions with M. Virions accumulate in large, smooth-walled vesicles that are exocytotically released from cells [4].

Despite lacking a significant amino acid sequence homology, SARS-CoV M shares structural and functional similarities with other coronavirus M proteins [27]. In addition to having an amino-terminal ectodomain, a triple-membrane spanning domain, and a carboxyl-terminal endodomain [19], [28], coronavirus M proteins localize exclusively in the ER/Golgi area [29], [30], [31]. However, the M proteins of SARS-CoV, the transmissible gastroenteritis virus, and the feline infectious peritonitis virus are all capable of reaching the plasma membrane [32], [33], [34], [35].

M plus E [36], [37], [38] or M plus N [16], [39] are minimum requirements for SARS-CoV VLP formation, and the combined expression of M, N and E is necessary for efficient VLP production [40]. SARS-CoV M has been detected in medium when expressed alone [37]. We previously demonstrated that SARS-CoV M is capable of self-association and secretion into medium as membrane-enveloped vesicles with a buoyant density slightly less than that of VLPs formed by M plus N [41]. Since N is undetectable in medium without M coexpression, it appears that SARS-CoV M directs VLP assembly by incorporating N into VLPs. Accordingly, mutations that block SARS-CoV M self-assembly or secretion also block VLP assembly, regardless of their effect (or lack of) on M-N interaction.

Our goal in this study was to identify specific SARS-CoV M amino acid residues that are critical for VLP assembly. Site-directed mutagenesis results suggest the involvement of M cytoplasmic tail dileucine residues in the packaging of N into VLPs. We observed that amino acid residues that are important for M self-assembly or secretion are dispersed along the carboxyl-terminal endodomain and the amino-terminal region, including the transmembrane domains. This finding supports the proposal that multiple SARS-CoV M regions are involved in M self-assembly. Here we will report on our identification of several amino acid residues that may play a role in SARS-CoV assembly.

## Results

### SARS-CoV M Cysteine Residues are Not Proximally Located at the Dimer Interface

**Figure 1:**
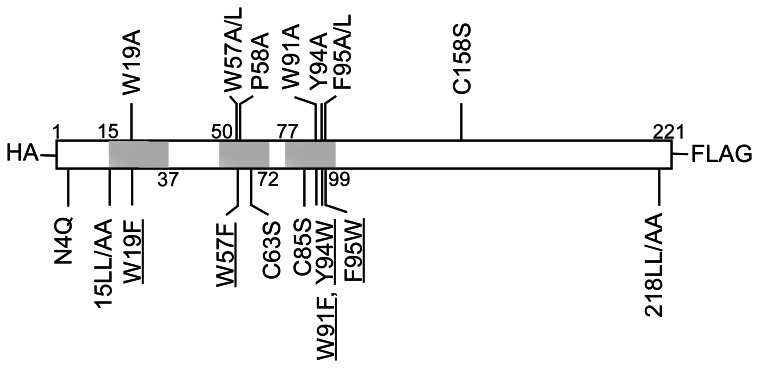
Schematic representations of SARS-CoV M mutations. Wild-type (wt) SARS-CoV M protein is shown with three predicted transmembrane domains (shaded boxes). Amino acid substitutions at M codon positions are indicated. An HA or FLAG epitope tagged at the amino or carboxyl terminus is designated as HA-M and M-FLAG, respectively. W57A/L and F95A/L indicate an Ala or Leu substitution at W57 and F95. Alanine substitutions of the di-leucine motif at codons 15–16 and 218–219 are designated as 15LL/AA and 218LL/AA, respectively. Underlined mutations denote that changing the aromatic residue to Ala or Leu markedly affected M secretion, but replacement with another aromatic residue did not. The ability for each construct to release or produce VLPs with coexpressed N is summarized in Table 1.

**Figure 2:**
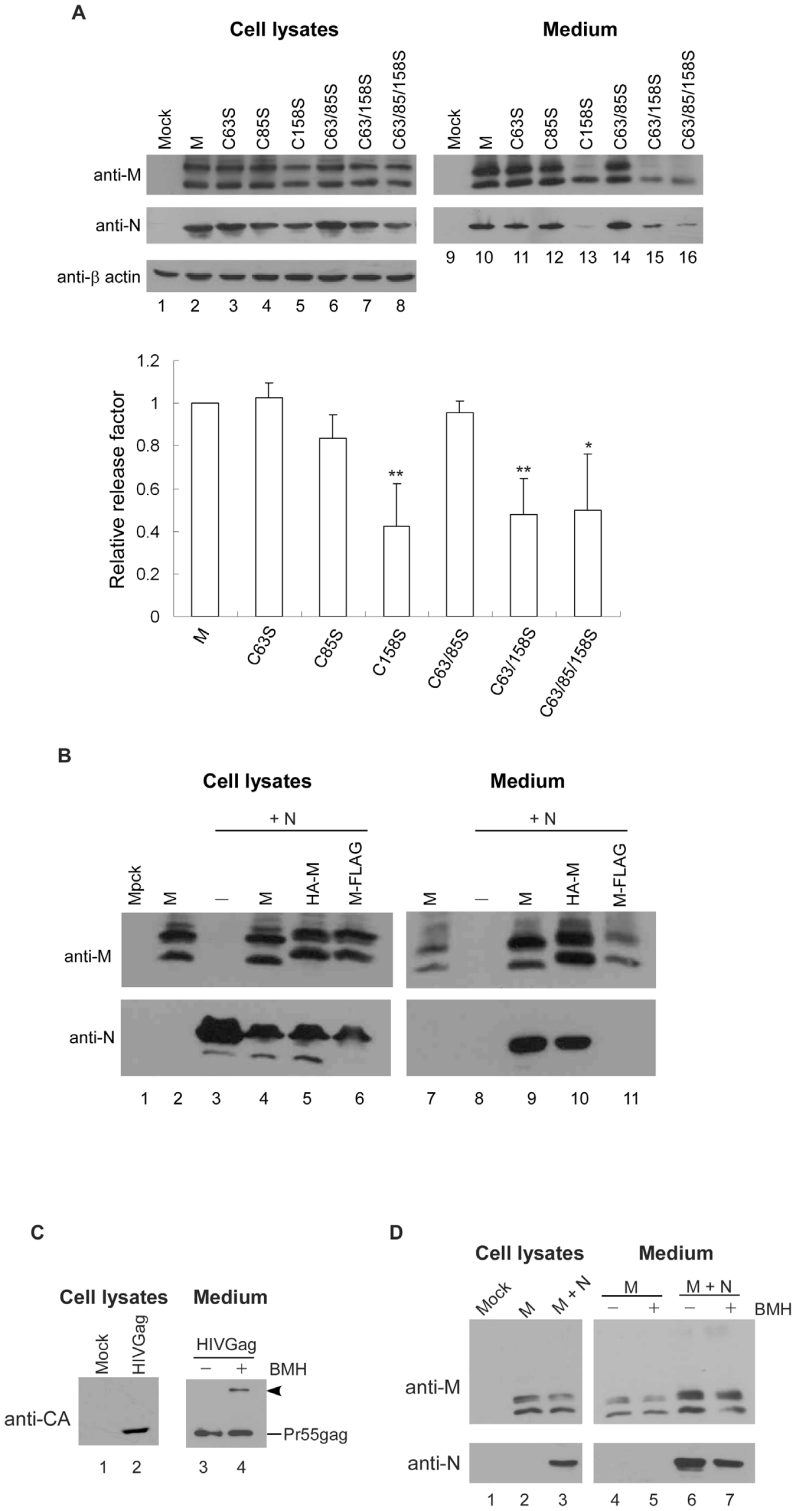
Mutational effects on M protein release and VLP assembly. (A) Results from analysis of SARS-CoV M cysteine residues in VLP assembly. (A) 293T cells were cotransfected with SARS-CoV N and indicated wt or mutant M expression vector. C63/85S, C63/158S and C63/85/158S designate combined double or triple alanine substitutions in cysteine residues 63, 85 and 158. At 24 to 36 h post-transfection, supernatants and cells were collected and prepared for protein analysis. Medium pellet samples corresponding to 50% of total and cell lysate samples corresponding to 5% of total were fractionated by 10% SDS-PAGE and electroblotted onto nitrocellulose filters. SARS-CoV M was probed with rabbit antiserum, and N was detected with a mouse anti-N monoclonal antibody. M proteins in medium or cell samples were quantified by scanning mutant and wt M band densities from immunoblots. Ratios of M levels in medium to those in cells were determined for each mutant and normalized to wt medium/cell ratios in parallel experiments. Error bars indicate standard deviations. *, *p<*0.05; **, *p<*0.01. (B) A FLAG tagged at the M carboxyl terminus prevents VLP assembly. 293T cells were transfect with SARS-CoV N alone or together with the indicated wt or mutant M expression vector. At 24 to 36 h post-transfection, supernatants and cells were collected and prepared for protein analysis as described above. (C–D) Results from cross-linking analyses of SARS-CoV M and HIV-1 Gag proteins. Extracellular particles isolated from 293T culture supernatants expressing SARS-CoV M, M plus N, or HIV-1 Gag were mock-treated or treated with the cysteine-specific cross-linking chemical BMH (panel C, lane 4 and panel D, lanes 5 and 7) as described in Materials and Methods. Samples were subjected to Western immunoblotting following 1 h incubation at room temperature. Arrowhead indicates Pr55gag dimer position.

SARS-CoV M contains three cysteine residues: C63 and C85 are found at the second and third transmembrane domains, respectively, and C158 is located at the carboxyl-terminal endodomain (Fig. 1). Results from our tests to determine whether cysteine residues play a role in SARS-CoV VLP assembly indicate that a serine substitution at C63 or C85, or a combined C63/85S double-mutation, did not significantly affect VLP assembly and release (Fig. 2A, lanes 11, 12 and 14). In contrast, single or combined double or triple substitutions of cysteine residues involving C158 markedly affected VLP production, likely a result of reduced M secretion. Note that secreted M mutants carrying the C158S mutation are glycosylated form-deficient (Fig. 2A upper panel, lanes 13, 15 and 16), suggesting an M-C158S maturation defect via the classical secretory pathway (54). As a control, N expressed by itself was not released into medium (Fig. 2B, lane 8). An HA tagged at the M amino-terminus (HA-M) had no major effect on M release and N packaging; in contrast, a FLAG tagged at the M carboxyl terminus (M-FLAG) significantly affected N incorporation (Fig. 2B, lane 11). This is consistent with previously reported results [41]. We found that N coexpression slightly increased wt or secretion-competent M mutant release, but had little effect on the release of secretion-defective mutants. This observation does not alter our conclusion that N release or VLP production depends on the presence of secretion-competent M proteins. Since M+N VLP assembly is determined by M release capacity, we determined the mutational effects on M release capacity under N coexpression conditions. The results shown in Figure 2A suggest that C158 is necessary for SARS-CoV M self-assembly or release.

**Table 1:** Effects of M mutations on SARS-CoV VLP assembly.

Construct	Phenotype
HA-M, N4Q, 15LL/AA, W19F, W57F, C63S, C85S, W91F, Y94W, F95W	VLP assembly-competent
W19A, W57A/L, W91A, Y94A, F95A/L, C158S	M secretion-defective
M-FLAG, 218LL/AA	M secretion-competent, but defective in VLP production

To determine whether any of the three cysteine residues exist at the M dimer interface, we treated VLPs with bismaleimidohexane (BMH), a cysteine-specific cross-linking reagent. The HIV-1 precursor Pr55gag, which is capable of self-assembly into VLPs, served as a positive control. As shown in Figure 2C, we noted a band of approximately 110 kDa corresponding to the Pr55gag dimer (lane 4, arrowhead), which is consistent with an earlier report that cysteine residues in the Pr55gag interaction (I) domain are capable of cross-linking via BMH [42]. In contrast, we failed to detect dimeric or multimeric forms of M in repeat independent experiments (Fig. 2D, lanes 5 and 7). Combined, the data suggest that the C63, C85 and C158 residues found in the M dimer in a VLP context are not close enough to be cross-linked by BMH.

### Aromatic Residues at Specific SARS-CoV M Codons are Critical to VLP Assembly

While constructing the C85S mutation, we unintentionally created a triple mutant (C85S/F95L/S110G) that was severely defective in VLP assembly. Results from further analysis indicate that either the F95L or S110G mutation significantly affected M secretion and VLP production (data not shown). Since the S110 mutation is located in the highly conserved 107-SWWSFNPE-114 motif, it is not surprising that S110G impaired VLP assembly. When analyzing the impacts of a F95L mutation, we found that an alanine substitution for F95 significantly impaired VLP assembly, but a tryptophan substitution did not (details given below). This indication of an important VLP assembly role for the conserved aromatic residue at codon 95 served as our motivation to investigate similar roles for the nearby residues W91 and Y94–two conserved aromatic residues that co-reside with F95 in the third transmembrane domain. We also created alanine and leucine substitutions for W57, which is located in the second transmembrane domain (Fig. 1). P58, which is conserved and located next to W57, was changed into alanine because both tryptophan and proline may play a role in protein-protein interaction [43], [44]. Based on one research team’s proposal that the dileucine motif is involved in sorting and trafficking [45], we replaced two dileucine motifs located in the carboxyl-terminal (L218-L219) region with alanine. Based on past results suggesting that the M self-association domain is largely located among 50 amino-terminal residues [41], we changed an amino-terminal dileucine motif (L15–L16) and the W19 conserved aromatic residue into alanines to determine whether they are involved in VLP assembly.

**Figure 3:**
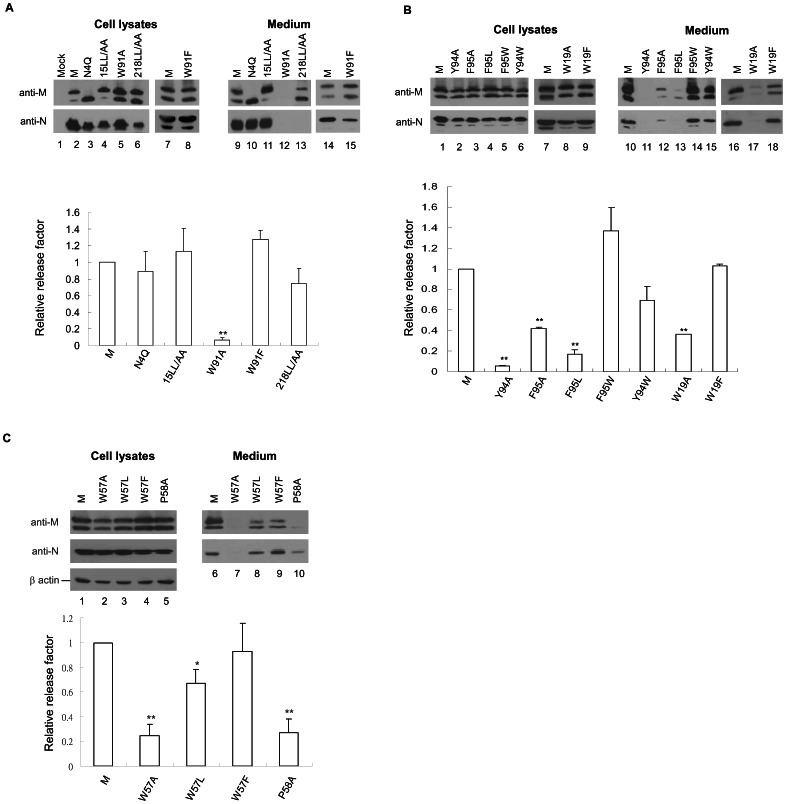
Effects of substitution mutations on SARS-CoV M secretion or VLP assembly. 293T cells were transfected with the indicated SARS-CoV M construct plus SARS-CoV N expression vector. At 24 to 36 h post-transfection, cells and supernatants were collected, prepared, and subjected to Western immunoblot analyses. M proteins were quantified and mutant M medium/cell ratios were normalized to those of wt M in parallel experiments as described in the Figure 2 caption. Blots are representative of three independent experiments. Error bars indicate standard deviations. *, *p*<0.05; **, *p*<0.01.

For an additional control we used the N-linked glycosylation blocking mutation N4Q, which is known for not exerting any major impacts on virus assembly or M trafficking [35], [46]. As expected, unglycosylated N4Q was capable of producing VLPs at near-wt levels (Fig. 3A, lanes 9 versus 10). The dileucine mutation 15LL/AA did not significantly affect M secretion or N packaging (Fig. 3A, lane 11). M-15LL/AA was almost found in glycosylated form (Fig. 3A, lanes 3 and 11). A possible explanation is that the 15LL/AA mutation may facilitate the M glycosylation process. The glycosylated form of M was occasionally (and predominantly) detected in medium (Fig. 3B, lane 16); this is insufficient evidence to confirm that N-glycan modification makes a significant contribution to M secretion or VLP assembly, since M-N4Q devoid of N-glycosylation is also competent in self-secretion and N packaging.

Substitution mutations at the M carboxyl tail (218LL/AA) resulted in a statistically insignificant decrease in M secretion, with coexpressed N barely detectable in medium (Fig. 3A, lane 13). This suggests that the 218LL/AA mutation may impair M-N association, which would agree with previous reports that the SARS-CoV M carboxyl-terminal region is involved in M-N interaction [16], [47]. Alanine or leucine changes in W19 (Fig. 3B, lane 17), W57 (Fig. 3C, lane 7), W91 (Fig. 3A, lane 12), Y94 or F95 (Fig. 3B, lanes 11–13) led to significant reductions in VLP production, likely due to defects in M secretion. P58 replacement with alanine also resulted in markedly impaired VLP assembly (Fig. 3C, lane 10). Our finding of detectable P58A-associated N in medium was likely due, at least in part, to longer immunoblot exposure (Fig. 3C lower panel, lane 10). Note that a phenylalanine replacement for W19, W57 or W91, or a tryptophan substitution for Y94 or F95 did not significantly impact VLP assembly (Fig. 3A, line 15; Fig. 3B, lanes 14, 15 and 18; Fig. 3C, lane 9). These data suggest that the retention of an aromatic amino acid residue at M codons 19, 57, 91, 94 or 95 is critical for SARS-CoV VLP assembly.

### Secretion-defective M Mutants are Still Capable of Self-association or Association with wt M

**Figure 4:**
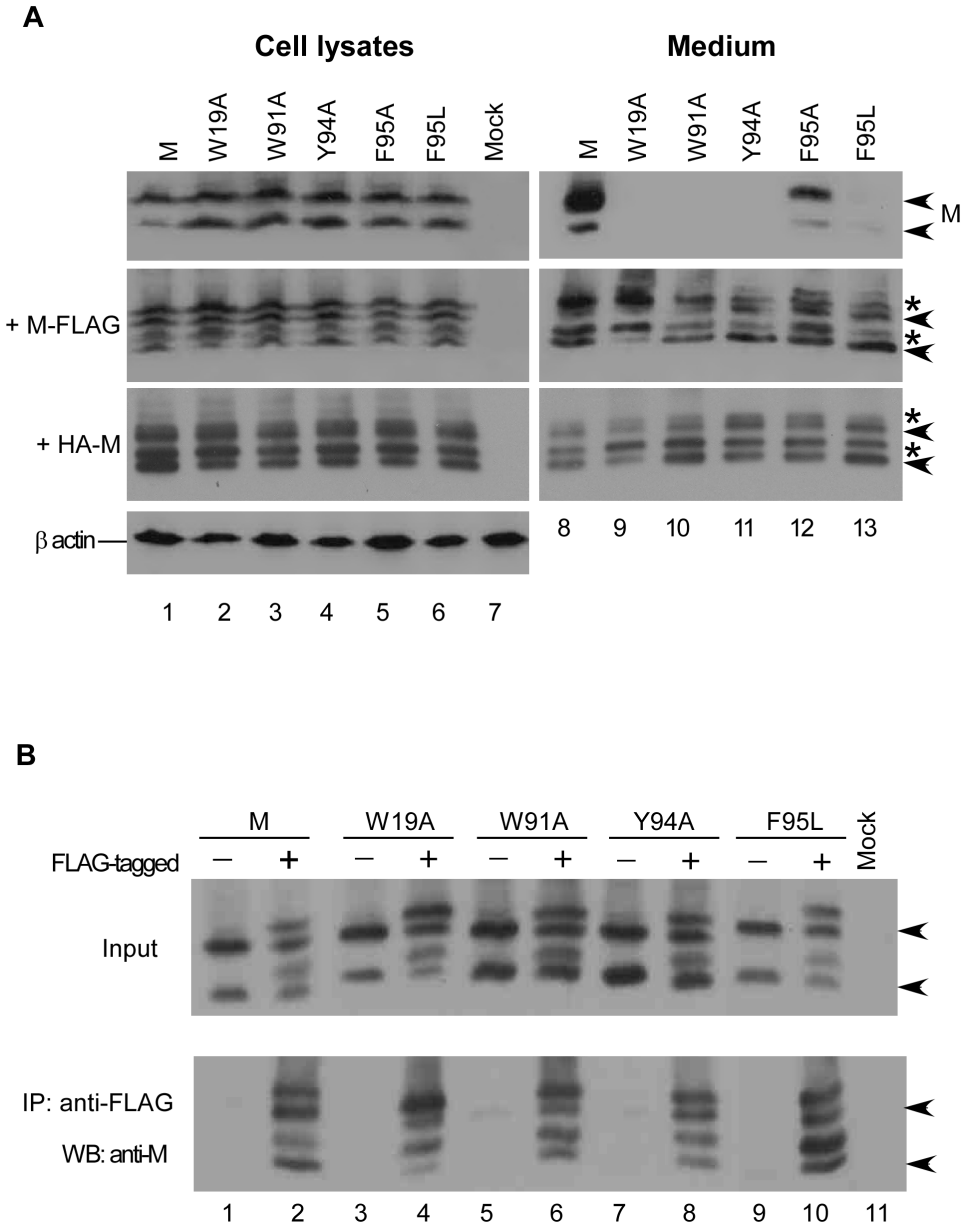
Effects of substitution mutations on M-M interaction. (A) Associations between mutant M proteins and FLAG-tagged or HA-tagged wt M. 293T cells were transfected with the indicated plasmid alone (upper panels) or with a FLAG-tagged (M-FLAG) or HA-tagged (HA-M) SARS-CoV M expression vector. At 24–36 h post-transfection, cells and medium were harvested and subjected to Western immunoblot analyses as described in the Figure 2 caption. Arrowheads and asterisks denote M and FLAG-tagged M positions, respectively. (B) Co-immunoprecipitation of M with FLAG-tagged M. 293T cells were transfected with the SCoV M wt or indicated mutant plasmid alone or with its FLAG-tagged counterpart (lanes 2, 4, 6, 8 and 10). Cell lysates were subjected to Western immunoblotting 48 h post-transfection. Equal amounts of cell lysates were mixed with anti-FLAG affinity gel and incubated for 2 h at 4°C. Bead-bound complexes were pelleted, washed, and subjected to Western immunoblotting.

Since M-M interaction involves multiple M sequence regions, we predicted that secretion-defective mutants associated with wt M would be released into medium. To test this idea, we coexpressed each of the secretion-defective mutants with M-FLAG or HA-M. W91A, Y94A and F95L release levels were noticeably increased following M-FLAG or HA-M coexpression (Fig. 4A, lanes 10, 11 and 13). Although W19A was barely detectable in medium with coexpressed M-FLAG, HA-M coexpression resulted in readily detectable W19A in medium (Fig. 4A, lane 9), possibly due in part to more efficient association with HA-M than with M-FLAG. Note the higher level of F95L compared to M-FLAG and HA-M in medium when F95L was coexpressed with M-FLAG or HA-M (Fig. 4A, lanes 13). We have tried to find an adequate explanation for this observation. It may be that the released chimeric vesicles contain greater amounts of F95L than of HA-M or M-FLAG due to favorable F95L incorporation. We observed that in the presence of M-FLAG or HA-M, immature unglycosylated forms of W91A, Y94A and F95L were more abundant in medium than their glycosylated counterparts, while F95A had a greater abundance of the unglycosylated form in medium when coexpressed with HA-M (Fig. 4A, lanes 10–13, lower vs. upper arrowheads). This is likely evidence of preferential association between HA-M or M-FLAG and immature unglycosylated mutant forms. Combined, these results suggest that some secretion-defective mutants can be rescued into wt M particles via M-M interaction.

We performed co-immunoprecipitation experiments to further determine whether reduced M secretion was due to a self-association defect. First, we individually coexpressed secretion-defective mutants with their FLAG-tagged counterparts. As shown in Figure 4B, W19A, W91A, Y94A or F95L were coprecipitated with their FLAG-tagged versions. According to velocity sedimentation analyses of cell lysates containing expressed M proteins, W19A, W91A, Y94A and F95A/L were capable of multimerizing into high-molecular-weight complexes in a pattern that was difficult to distinguish from that of the wt M (data not shown). These results suggest that secretion-defective mutants are capable of a certain level of self-association despite defective VLP assembly or release.

### Delayed M Secretion and Reduced Mutant VLP Production

Results from immunofluorescence studies indicate that wt or secretion-competent mutants are localized in both perinuclear and plasma membrane areas. Although most of the secretion-defective mutants did not show plasma membrane localization, the secretion-defective W19A was found in both plasma membrane and perinuclear areas–a staining pattern indistinguishable from that of the wt (data not shown). This suggests that the M release defect is not completely attributable to a defect in plasma membrane localization.

**Figure 5:**
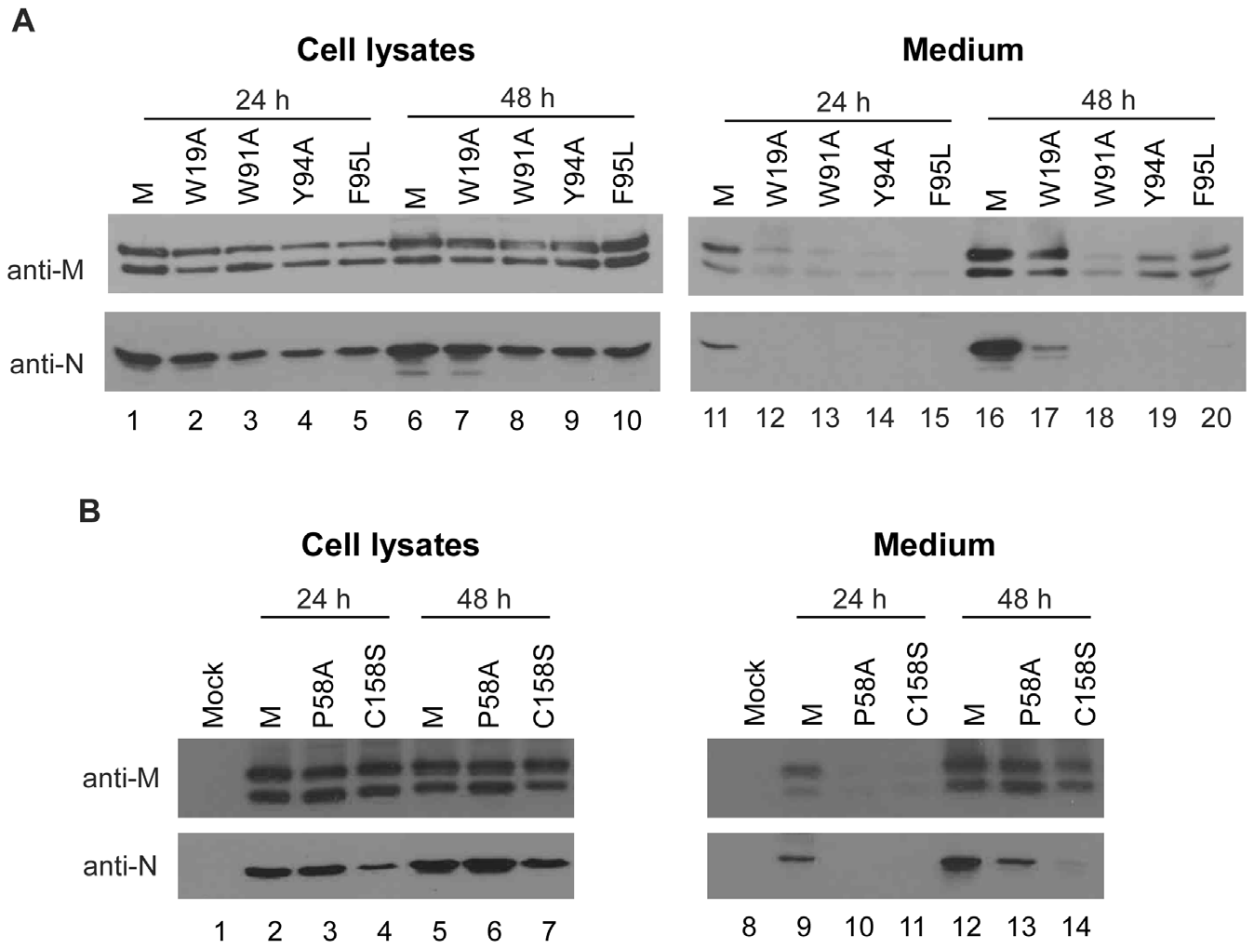
VLP accumulation in medium. 293T cells were cotransfected with SARS-CoV N and wt or indicated mutant M expression vector. Equal amounts of cells were placed on two dish plates 4 h post-transfection. Supernatant and cells were harvested at 24 h and 48 h post-transfection, prepared, and subjected to Western immunoblotting.

We predicted that in cases where mutants express delayed assembly or budding, medium VLP quantities would increase as incubation time increased. Our data indicate that most of the secretion-defective mutants were readily detectable in culture supernatant 48 h post-transfection (Fig. 5A, lanes 17–20 and Fig. 5B, lanes 12–14). This finding suggests that reductions in M secretion or VLP production are partly due to delays in assembly or budding. However, after 48 h of incubation, N remained barely detectable in medium, or detectable but not equivalent to the level of released M, suggesting a mutant defect in terms of N packaging. This finding also suggests that the M mutations may have affected N viral incorporation in addition to impairing M secretion.

### The M Carboxyl-terminal Tail Domain Plays a Critical Role in Determining N Viral Incorporation

**Figure 6:**
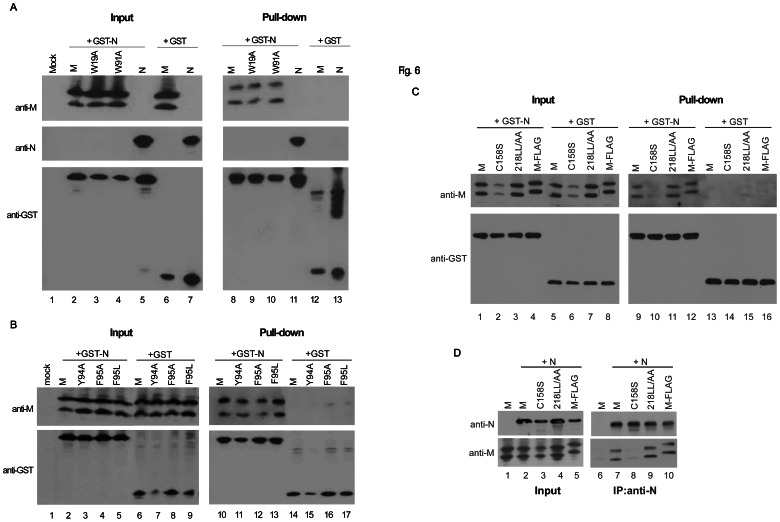
Co-precipitation of M mutants with SARS-CoV N. (A–C) GST pull-down assay. 293T cells were cotransfected with GST-N (SARS-CoV N fused to the GST carboxyl terminus) and N, wt or indicated M mutant plasmid. Aliquots of cell lysates preceding and flowing GST pull-down were subjected to Western immunoblotting using anti-GST, anti-M and anti-N antibodies as probes. (D) Co-immunoprecipitation assay. 293T cells were cotransfected with SARS-CoV N with the indicated wt or mutant M plasmid. Aliquots of cell lysates were subjected to co-immunoprecipitation with an anti-N monoclonal antibody.

Next, we investigated whether secretion-defective M mutants can still interact with N, and whether the failure of secretion-competent 218LL/AA to form VLPs is due to a defect in N association. N, wt or mutant M was coexpressed with GST-N (a GST fused to the N amino-terminus) and subjected to GST-pull down assays. Since N contains a dimerization domain, N association with GST-N was used as a control [48], [49]. As expected, N was efficiently pulled down by GST-N. With the exception of C158S, all of the tested secretion-defective M mutants were co-pulled down with GST-N (Fig. 6), suggesting that M mutants are still capable of N association despite being defective in terms of cell release. Unexpectedly, 218LL/AA and M-FLAG were efficiently pulled down by GST-N (Fig. 6C, lanes 11 and 12). Similar results were obtained from co-immunoprecipitation experiments using an anti-N antibody (Fig. 6D). Levels of N-associated M-C158S, as determined by GST pull-down or co-immunoprecipitation assays, were lower compared to those of the other mutants used in this study (Fig. 6C, lane 10 and Fig. 6D, lane 8). These data suggest that the C158S mutation significantly affected M-N interaction, and that the 218LL/AA and M-FLAG mutations at the M carboxyl-terminal tail prevented N from viral incorporation during M-directed virion morphogenesis, even though they did not significantly affect M-N interaction.

**Figure 7:**
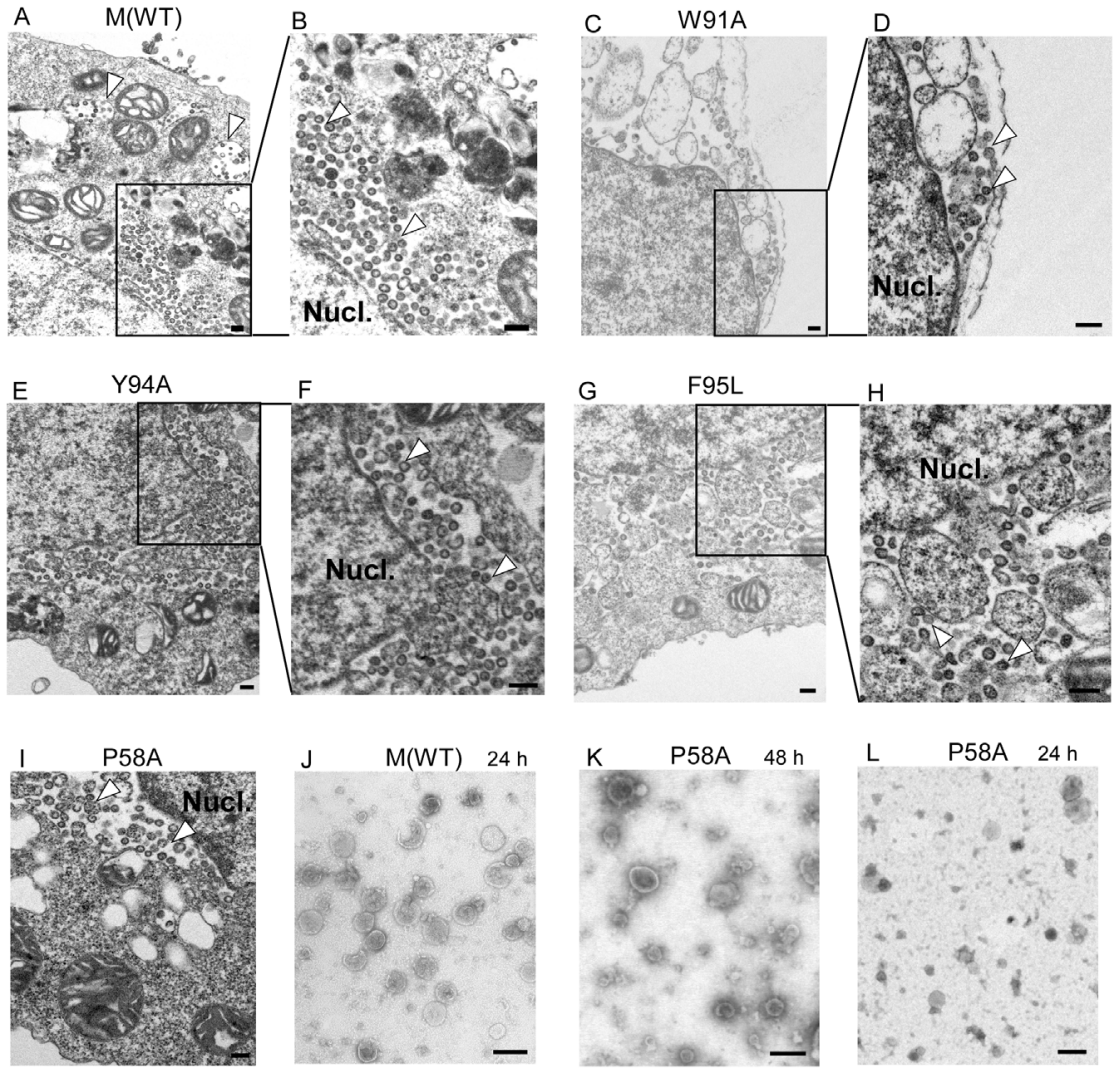
SARS-CoV VLP analysis. 293T cells were cotransfected with SARS-CoV N and wt or indicated M mutant plasmid. At 48 h post-transfection, cells and supernatants were collected. Cells (panels A to I) were fixed and prepared for electron microscopy analysis as described in Material and Methods. Supernatants (panels J to L) were filtered and pelleted through 20% sucrose cushions, resuspended in PBS buffer and stained. Cell and supernatant samples were observed with a transmission electron microscope. The high-power view in the inset shows VLPs near cell nuclei (arrowheads). Bars, 100 nm.

To determine whether the intracellular association between mutant M and N leads to VLP formation, cells coexpressing N and either wt or mutant M were observed with a transmission electron microscope (TEM). As expected, numerous VLPs were observed localized in the perinuclear areas of cells coexpressing wt M and N (Fig. 7, panels A and B), which is consistent with a previous report [16]. Intracytoplasmic vesicles containing VLPs were also observed (Fig. 7A, arrowheads). Further, we noted VLPs near the nuclei of cells coexpressing N and secretion-defective W91A (Fig. 7D). VLPs were also detectable in cells coexpressing N and Y94A, F95L or P58A M mutants (Figs. 7E–7I). Figure 5 shows culture supernatants collected from cotransfectants 24 or 48 h post-transfection. At 24 h, spherical particles approximately 100 nm in diameter were observed in wt M and N cotransfectant samples (Fig. 7J), which is consistent with previous results. In contrast, VLPs from cells coexpressing N and P58A were undetectable or barely detectable until 48 h post-transfection (Fig. 7K). Also at 24 h post-transfection, medium samples containing P58A plus N cotransfectants were almost identical to the mock-transfected sample–that is, we detected some vesicles, but no VLPs (Fig. 7L). VLPs were barely detectable in culture supernatants derived from cells expressing N plus W91A, Y94A, F95L/A, C158S or 218LL/AA. No VLPs were found in cells coexpressing 218LL/AA plus N, and no VLPs were detected during TEM observations of concentrated gradient fractions of cell lysates containing 218LL/AA and N. In contrast, all other secretion-defective M mutants were still capable of associating with N and subsequently forming some intracellular VLPs, despite being VLP release-defective.

## Discussion

According to our results, the effects of SARS-CoV M mutations on VLP assembly are largely determined by the capability of M mutants to be released from cells. According to Siu et al. [40], M expressed alone is barely secreted, and N coexpression is required for efficient M release from Vero E6 cells [40]. This observation may be due to different expression systems used in the two studies. It could be that 293T cells release and/or express SARS-CoV membrane proteins more efficiently than Vero E6 cells. This may partly explain why 293T cells produce VLP-associated spike (S) proteins at levels 28-fold higher than Vero E6 cells, as reported by Siu et al. [40]. Nevertheless, the capability of SARS-CoV M to self-assemble and release or to form VLPs with coexpressed N represents a convenient strategy for determining M-M and M-N interaction domains.

M-C158S is defective in both secretion and N association, suggesting that C158 residue is important for SARS-CoV assembly. Although we did not detect any intermolecular disulfide linkages, there is still a possibility of intramolecular disulfide bonds occurring during M self-assembly or secretion, which would partly explain why the C158S mutation exerts a significant effect on M release. Evidence showing that secretion-defective SARS-CoV M mutants are capable of self-association or association with wt M supports the proposal that multiple M regions are involved in self-association [52].

The inability of the 218LL/AA or M-FLAG mutant to package N suggests that the M carboxyl-terminal tail domain is responsible for M-N interaction. At least three research teams have suggested that the coronavirus M carboxyl tail region is important for N association [26], [47], [50]. However, our GST pull-down and co-immunoprecipitation experiment results suggest that alanine substitutions for the highly conserved dileucine motif 218-LL-219 failed to significantly impact SARS-CoV M-N interaction; this finding agrees with data from yeast two-hybrid and surface plasmon resonance (SPR) assays [47]. Nal et al. [54] have proposed that SARS-CoV M recycles to Golgi complexes via endocytosis once it reaches the plasma membrane. Accordingly, substitution mutations at 218-LL-219, or a FLAG tagged at the carboxyl-terminus, may block M sorting or trafficking to the Golgi area, resulting in defective VLP formation. This scenario may partly explain why M-FLAG and 218LL/AA were capable of N association following the disruption of cellular compartments.

The M carboxyl-terminal tail is also important for MHV assembly: the deletion of a single amino acid residue from the M carboxyl terminus acts as a significant barrier to VLP assembly [51], [52]. MHV VLP formation is dependent on coexpression with the E protein [10], [52]. Accordingly, impaired MHV VLP assembly due to a M carboxyl-terminal mutation is largely the result of a defect in M-E interaction [52], whereas SARS-CoV M carboxyl-terminal mutations such as 218LL/AA and M-FLAG do not affect M self-assembly and release. In a previous study we observed that SARS-CoV E is also secretable into culture medium (unpublished results); this is in agreement with a report that levels of SARS-CoV VLPs formed by M plus N noticeably increased following E coexpression [40]. However, E coexpression did not significantly enhance the VLP yields of SARS-CoV M mutants (data not shown), suggesting that E is incapable of compensating for M mutants in terms of directing VLP assembly.

With the exception of C158, we found that all of the identified amino acid residues deemed important for SARS-CoV M self-assembly were either proline or aromatic (e.g., tryptophan, phenylalanine or tyrosine). Aromatic residues have been shown to mediate the self-assembly of different soluble proteins via <pi>-<pi> interactions between polar aromatic rings [55], [56], [57]. Aromatic side chains have been proposed as favoring intra- and inter-peptide electrostatic interaction contributing to protein secondary structure and stable protein-protein interaction [58]. One research team has demonstrated that an aromatic-X-X-aromatic motif located in the transmembrane (TM) domain of EpsM (a cholera toxin secretion protein) is essential for stabilizing TM dimerization [59]. It is likely that the SARS-CoV M aromatic-XX-aromatic motif (91-WXXY-94), which resides in the predictive second TM domain, serves a similar function in stabilizing M dimerization. In addition, a more recent study suggests that coronavirus M is capable of adopting two conformations associated with membrane curvature regulation [53]. Accordingly, the replacement of conserved aromatic residues with alanine or leucine may disrupt M conversion from one form to another, resulting in a membrane-bending defect. This scenario may partly account for decreased mutant VLP yields.

It remains to be determined whether the other M aromatic residues are important for SARS-CoV assembly. Our preliminary study found that an alanine substitution at the highly conserved W54 exerted no detectable effect on SARS-CoV VLP assembly, suggesting that some conserved aromatic residues are not involved in that process. The replacement of SARS-CoV M codons W19, W57, W91, Y94 or F95 with other aromatic residues did not exert detrimental effects on VLP assembly. A future task is to determine if the same is true for other M aromatic residues.

## Materials and Methods

### Plasmid Construction

Codon optimized SARS-CoV M and N expression vectors were provided by G. J. Nabel [16]. A pair of upstream and downstream primers was used to amplify M-coding fragments via PCR-based overlap extension mutagenesis [60] with the SARS-CoV M expression vector serving as a template: the 5′-GTCTGAGCAGTACTCGTTGCTG-3 forward primer (referred to as the N primer) and the 5′- GGAAAGGACAGTGGGAGTGGCAC-3′ reverse primer. Oligonucleotide primers containing the substitution mutation codons were available on request. Purified PCR product was digested with BamHI and EcoRV and ligated into the SARS-CoV M expression vector. GST-N or HIV-1 Gag expression vector has been described elsewhere [61].

### Cell Culture and Transfection

293T cells and HeLa were maintained in Dulbecco’s modified Eagle’s medium (DMEM) supplemented with 10% fetal calf serum (GIBCO). Confluent cells were trypsinized and split 1∶10 onto 10 cm dishes 24 h prior to transfection. For each construct, cells were transfected with 10 µg of plasmid DNA using the calcium phosphate precipitation method; 50 µm chloroquine was added to enhance transfection efficiency. Unless otherwise indicated, 5 µg of each plasmid was used for co-transfection.

### Western Immunoblot

At 24–48 h post-transfection, supernatant from transfected cells was collected, filtered, and centrifuged through 2 ml of 20% sucrose in TSE (10 mM Tris-HCl [pH 7.5], 100 mM NaCl, 1 mM EDTA plus 0.1 mM phenylmethylsulfonyl fluoride [PMSF]) at 4°C for 40 min at 274,000×*g*. Pellets were suspended in IPB (20 mM Tris-HCl [pH 7.5], 150 mM NaCl, 1 mM EDTA, 0.1% SDS, 0.5% sodium deoxycholate, 1% Triton X-100, 0.02% sodium azide) plus 0.1 mM PMSF. Cells were rinsed with ice-cold phosphate-buffered saline (PBS), collected in IPB plus 0.1 mM PMSF, and microcentrifuged at 4°C for 15 min at 13,700×*g* to remove unbroken cells and debris. Supernatant and cell samples were mixed with equal volumes of 2X sample buffer (12.5 mM Tris-HCl [pH 6.8], 2% SDS, 20% glycerol, 0.25% bromphenol blue) and 5% β-mercaptoethanol and boiled for 5 min or (for the M-containing samples) incubated at 45°C for 10 min. Samples were resolved by electrophoresis on SDS-polyacrylamide gels and electroblotted onto nitrocellulose membranes. Membrane-bound M and M-FLAG proteins were immunodetected using a SARS-CoV M rabbit anitserum (Rockland). For SARS-CoV N detection, a mouse monoclonal antibody [62] was used at a dilution of 1∶5,000. The secondary antibody was a sheep anti-mouse or donkey anti-rabbit horseradish peroxidase-(HRP) conjugated antibody (Invitrogen), both at 1∶5,000 dilutions.

### Laser Scanning Immunofluorescence Microscopy

Confluent HeLa cells were split 1∶80 onto coverslips 24 h before transfection. At 24 h post-transfection, cells were washed with PBS and permeabilized at room temperature for 10 min in PBS plus 0.1% Triton X-100 following fixation at 4°C for 20 min with 3.7% formaldehyde. Samples were incubated with a rabbit anti-SARS-CoV M or with a mouse anti-N monoclonal antibody at a dilution of 1∶1000 for 1 h. A goat anti-rabbit rhodamine-conjugated antibody or a rabbit anti-mouse fluorescein isothiocyanate-conjugated antibody (Cappel, ICN Pharmaceuticals, Aurora, OH) at a 1∶100 dilution for 30 min. Following each incubation, samples were subjected to three washes (5 to 10 min each) with DMEM/calf serum. After a final DMEM/calf serum wash, the coverslips were washed three times with PBS and mounted in 50% glycerol in PBS for viewing. Images were analyzed and photographs taken using the inverted laser Zeiss Axiovert 200 M microscope.

### Velocity Sedimentation Analysis of Cytoplasmic M Proteins

Cells were rinsed twice with PBS, pelleted and resuspended in 1 ml TEN buffer (10 mM Tris-HCl [pH 7.4], 1 mM EDTA, 100 mM NaCl) containing Complete protease inhibitor cocktail followed by homogenization using a sonicator. The cell lysates then were centrifuged at 3,000 rpm for 20 min at 4°C. Five hundred µl of the postnuclear supernatants were mixed with an equal amount of TEN buffer, and were then applied to the top of a pre-made 25–45% discontinuous sucrose gradient. This gradient was prepared in TEN buffer containing 1 ml of each of 25%, 35%, and 45% sucrose. The gradient was then centrifuged at 130,000 × *g* for 1 hour at 4°C. Five 0.8-ml fractions were collected from the top of the centrifuge tubes. The proteins present in aliquots of each fraction were precipitated with 10% TCA and subjected to western blot analysis as described in the membrane flotation assay.

### FLAG Fusion Protein Immunoprecipitation

293T cells transfected with FLAG-tagged M expression vector were collected in lysis buffer (50 mM Tris-HCl [pH 7.4], 150 mM NaCl, 1 mM EDTA, 1% Triton X-100) containing complete protease inhibitor cocktail (Roche) and microcentrifuged at 4°C for 15 min at 13,700×*g* (14,000 rpm) to remove unbroken cells and debris. Aliquots of post-nuclear supernatant (PNS) were mixed with equal amounts of 2X sample buffer and held for Western blot analysis. Lysis buffer was added to the remaining PNS samples to final volumes of 500 µl, and each sample was mixed with 20 µl of anti-FLAG affinity gel (Sigma). All reactions took place at 4°C overnight on a rocking mixer. Immunoprecipitate-associated resin or bead-bound complexes were pelleted, washed tree times with lysis buffer, two times with PBS, eluted with 1X sample buffer, and subjected to SDS-10% PAGE as described above.

### Cross-linking Methods

Cross-linking reagent bis-maleido hexame (BMH; Pierce) was prepared in dimethyl sulfoxide (DMSO) as a 20 mM solution. Virus-like particles were prepared in PBS and aliquoted at 20-µl fractions that mock-treated with 1 µl DMSO or treated with 1 µl of 20 mM BMH in DMSO. Reaction mixtures were vortexed gently and incubated for 1 h at room temperature. Samples were mixed with equal volumes of 2X sample buffer and 5% β-mercaptoethanol and incubated at 45°C for 10 min prior to electrophoresis.

### Electron Microscopy

Cells were harvested 24 h post-transfection and fixed in 0.1 M Cacodylate buffer containing 2.5% glutaraldehyde, post-fixed with 1% osium tetroxide, dehydrated in ethanol and embedded in Spurr resin. Thin section was cut with an ultramicrotome, stained with 5% uranyl acetate and 0.4% lead citrate. Concentrated viral samples were placed onto carbon-coated, UV-treated 200 mesh copper grids for 2 min. Sample-containing grids were rinsed for 15 secs in water, dried with filter paper, and stained for 1 min in filtered 1.3% uranyl acetate. Excess staining solution was removed by applying filter paper to the edge of each grid. Grids were allowed to dry before viewing with a JOEL JEM-2000 EXII transmission electron microscope. Images were collected at 20,000× and 60,000×.
